# A Study of Factors Associated With Carrying Angle of the Human Elbow in Pediatric Age Group

**DOI:** 10.7759/cureus.25478

**Published:** 2022-05-30

**Authors:** Narendra Singh Kushwaha, Vikas Verma, Arpit Singh, Yashvardhan Sharma, Ajai Singh

**Affiliations:** 1 Orthopedic Surgery, King George's Medical University (KGMU), Lucknow, IND; 2 Pediatric Orthopedics, King George's Medical University (KGMU), Lucknow, IND

**Keywords:** morphometric, predictive factor, child, pediatric age group, elbow carrying angle

## Abstract

Purpose

This prospective cohort study aims to determine the factors that are associated with the carrying angle of the human elbow in the pediatric age group.

Methods

One hundred forty children up to 15 years of age were assessed for age, sex, forearm lengths of both sides, arm length of both sides, trans-trochanteric diameter, height, BMI, the inter-epicondylar distance of both sides, Baumann’s angle of both sides, presence or absence of secondary sexual characteristics, clinical carrying angle (CCA) of both sides, and radiological carrying angle (RCA) of both sides. Unpaired t-test was used to compare the means of carrying angle in the unrelated groups, namely gender and secondary sexual characteristics. The strength and direction of the relationship between carrying angle and continuous variables were tested by calculating Pearson’s correlation. Variables found to be associated with carrying angle at significance level >0.25 on bi-variable analysis were used to design a linear regression model to identify factors associated with carrying angle.

Results

The mean age was 5.84±4.76 years. Ninety-eight (70%) were males, and forty-two (30%) were females. The mean CCA on the right side was 8.55±2.01. The mean CCA on the left side was 8.77±2.03. The mean RCA on the right side was 8.85±2.09. The mean RCA on the left side was 9.07±2.13. On bi-variable analysis, the CCA was found to be associated with age, secondary sexual characteristics, weight, height, arm length, forearm length, inter-epicondylar distance, trans-trochanteric distance, and Baumann’s angle. CCA was found to be significantly negatively correlated with BMI. On multivariate linear regression, the CCA was found to be associated with age and inter-epicondylar distance.

Conclusion

Age and inter-epicondylar distance are the true associations of carrying angle.

## Introduction

When the elbow joint is fully extended and supinated, the forearm is not in a straight line with the arm; instead, it is laterally deflected, and an angle is formed between the long axis of the arm and the long axis of the forearm. This angle is referred to as the carrying angle of the elbow [1]. The carrying angle apparently develops as a response to pronation of the forearm and keeps the swinging upper extremity away from the side of the pelvis during walking [2]. Anatomically, it is explained by the most distal location of the trochlea compared to the capitulum in the humerus [3] and slight valgus angulation of the trochlear notch of the ulna with the shaft of the humerus [4]. 

In healthy children, the carrying angle and the range of motion at the elbow joint increase with age until skeletal maturity [5]. However, a study showed that clinical carrying angle (CCA) increases up to the age of 15 years, followed by a slight decrease in the angle [6]. The same study reported the increment rate per year in boys and girls to be 0.42 and 0.60, respectively [6]. In adults, the mean carrying angle for males and females are 10º and 13º, respectively [6]. An increase in the carrying angle during the growing age may lead to elbow instability [5,6], pain during exercise and throwing [7,8], decreased flexion at the elbow [9], increased chances of dislocation of the elbow [10], and increased chances of fracture of the distal humeral epiphysis [2]. In addition, a carrying angle of >15° has been reported to be a risk factor for non-traumatic ulnar neuropathy at the elbow [11].

Several studies have investigated the relationship between carrying angle and age [5,6], sex [6], dominant side [4,12], body characteristics such as trans-trochanteric diameter [4], height [12], length of the forearm [13], length of the arm [14] constitution [4], race, and inter-epicondylar distance [15]. A study done on healthy Chinese children reported a negative correlation between Baumann’s angle and carrying angle [16]. However, Baumann’s angle has been found to be an inaccurate indicator of carrying angle when treating displaced supracondylar fracture [17]. The effect of body characteristics on carrying angle at the elbow has not been extensively studied in the pediatric population. Therefore, we decided to study the effect of body characteristics on carrying angle in the pediatric population. The objectives of this study were to identify the factors associated with carrying angle and quantify the effect of these factors on the carrying angle.

## Materials and methods

This cross-sectional study was conducted on children up to 15 years. Written informed consent was obtained from the parents who volunteered for their children to be enrolled in the study. In children above seven years of age, assent was obtained from children and written informed consent from the parents. The institutional ethics committee approved the study (ref. code: 103rrd ECM II B-Thesis/P35). We excluded children with a history of trauma, tumor, bone or joint infections, surgery and burns to any extremity, children with post-polio residual paralysis, metabolic bone disorders, osteogenesis imperfecta, fibrous dysplasia, neurological conditions with abnormal muscle tone, arthritis, congenital orthopedic deformities, and those with ligamentous laxity.

The sample size was calculated to be 140 based on the maximum and minimum variation within groups and considering the number of study parameters to correlate using the formula below:

 n = k [(Zα​​ + Zβ) ( s1^2^ + s2^2^ )] / d^2^

Where s1 = 3.95, s2 = 3.37 the maximum and minimum SD within groups; k = 4, the design effect for considering number of study parameters to correlate [12]; d = min (s1,s2), the minimum mean difference consider to be clinically significant; type I error α = 5% corresponding to 95% confidence level; type II error β = 10% for detecting results with 90% power of the study

Children below the age of 15 years attending the outpatient clinic of the department of orthopedics were assessed for age, sex, forearm lengths of both sides, arm length of both sides, trans-trochanteric diameter, height, BMI, the inter-epicondylar distance of both sides, Baumann’s angle of both sides, presence or absence of secondary sexual characteristics, CCA of both sides, and radiological carrying angle (RCA) of both sides.

CCA was measured with the elbow in full extension and forearm in full supination using the manual goniometer. The CCA was measured as the angle between the central axis of the forearm and arm. The central axis of the forearm was taken as a line joining the midpoint of the inter-epicondylar line to the midpoint of the inter-styloid line at the wrist [13]. The central axis of the arm was taken as a line joining the midpoint of the inter-epicondylar line to the tip of the acromion process of the arm [13]. RCA was measured on a true anteroposterior (AP view of the X-ray of the elbow by measuring the angle between the longitudinal axis of the humeral shaft and the longitudinal axis of the shaft of the ulna [18-20]. The axis of the humerus and ulna were determined by joining at least two central points on the humerus and ulna [18-20]. Baumann’s angle was measured on a true AP view of the X-ray of the elbow. A straight line was drawn through the middle of the humeral shaft by taking two central points on the humeral shaft. A second line was drawn perpendicular to the humeral shaft. A third line was drawn along the lateral condylar physis. The angle between the first line and the third line was recorded as Baumann’s angle [21].

The technique described by Allouh MZ et al. [15] was used to measure the inter-epicondylar distance. The arm was lifted to shoulder level, and the forearm flexed to 90 degrees. This was done to make the humeral epicondyles prominent and easily palpable. A vernier caliper was used to measure the inter-epicondylar distance. The fixed arm of the caliper was placed on the lateral epicondyle, and the movable arm was then adjusted to the medial epicondyle. Trans-trochanteric diameter at the hip was measured with the subject in a standing position using a standard pelvimeter used by obstetricians [4]. The distance from the tip of the lateral epicondyle to the mid-point of a line joining radial and ulnar styloid was taken as the forearm's length. It was measured using a standard measuring tape with the arm flexed to 90º [16].

The data was collected on a Microsoft Excel sheet. SPSS was used for statistical analysis. Categorical variables were described using frequency tables or histograms. Continuous variables were described using measures of central tendency (mean, median, and mode) and measures of dispersion like SD.

Unpaired t-test was used to compare the means of carrying angle in the unrelated groups, namely gender and secondary sexual characteristics. The strength and direction of the relationship between carrying angle and continuous variables were tested by calculating Pearson's correlation coefficient. Variables found to be associated with carrying angle at significance level >0.25 on bivariable analysis were used to design a linear regression model to identify factors associated with carrying angle.

## Results

One hundred forty subjects were enrolled in the study. The mean age was 5.84±4.76 years. Ninety-eight (70%) were males, and 42 (30%) were females. Forty-six (32.9%) subjects were <2 years of age; 23 (16.4%) were >2-5 years of age; 44 (31.4%) were >5-10 years of age, and 27 (19.3%) were >10-15 years of age. The growth plate of the lateral condyle of the humerus was not visible in 20 subjects, and therefore Baumann's angle could be recorded in 120 subjects only.

The baseline characteristics of the subjects are shown in Tables 1-2. The means of CCA on the right and left sides were 8.55±2.01 and 8.77±2.03, respectively. The difference between the means of the right and the left side was found to be statistically insignificant (p=0.674). The RCA on the right and left sides were 8.85±2.09 and 9.07±2.13, respectively. The difference between the means of the right side and the left side was found to be statistically insignificant (p=0.654). Since the difference in carrying angles of the right and the left sides was insignificant, we calculated the average mean carrying angles by using the values of the two sides. In addition, averages of morphometric measures (length of arm, length of forearm, and inter-epicondylar distance) and Baumann's angle were calculated by using values for both sides. These were used for correlation and regression analysis to determine the factors associated with CCA. The mean CCA was calculated to be 8.66±2.02, and the mean RCA was calculated to be 8.99±2.11. The difference between CCA and RCA was found to be statistically insignificant (p=0.624). Since the difference between the means of CCA and RCA was not different, correlations and regression analysis were done for CCA alone.

**Table 1:** Baseline categorical characteristics.

Variable name		N	%
Gender	Male	98	70.0
	Female	42	30.0
Handedness	Right	128	91.4
	Left	12	8.6
Secondary sexual characteristics	Present	22	15.7
	Absent	118	84.3

**Table 2:** Quantitative characteristics of the enrolled subjects.

Variable name	Mean	SD	Median	Min	Max	Valid N
Age	5.84	4.76	6.00	.04	15.00	140
BMI	17.17	2.88	16.91	11.62	28.40	140
Mean arm length (cm)	18.39	5.67	19.95	9.10	29.80	140
Mean forearm length (cm)	15.65	5.51	16.80	7.00	25.80	140
Mean inter-epicondylar distance (cm)	5.78	1.61	6.40	2.80	8.10	140
Trans-trochanteric distance (cm)	28.47	7.54	30.40	13.20	40.80	140
Mean Baumann's angle	72.17	4.52	70.40	63.70	82.40	120
Clinical carrying angle	8.66	2.02	8.30	5.80	13.40	140
Radiological carrying angle	8.99	2.11	8.50	5.90	13.60	140

Bi-variable analysis

Unpaired t-test was applied to compare the values of mean CCAs in subjects with different sex, handedness, and secondary sexual characteristics.

The means of CCA in males and females were 8.61±2.01 and 8.94±2.01, respectively. The difference was statistically insignificant (p=0.641). The means of CCAs in those with secondary sexual characteristics and those without secondary sexual characteristics were 11.26±1.74 and 8.17±1.67, respectively. The difference was statistically significant (p<0.001). The means of CCA were found to be significantly different in different age groups (Table 3).

**Table 3:** Effect of age on the clinical carrying angle.

	Age intervals								P-value
	<2 years		2-5 years		>5-10 years		>10-15 years		
	Mean	SD	Mean	SD	Mean	SD	Mean	SD	
Clinical carrying angle	6.51	0.5	7.93	0.49	9.48	1.1	11.59	0.7	<0.001

CCA was found to have a significant positive correlation with age, weight, height, arm length, forearm length, inter-epicondylar distance, trans-trochanteric distance, and Baumann's angle. CCA was found to have a significant negative correlation with BMI (Table 4).

**Table 4:** Correlation of the clinical carrying angle with anthropometric measurements and Baumann's angle. **Correlation is significant at the 0.01 level (2 tailed). *Correlation is significant at the 0.05 level (2 tailed).

	Clinical carrying angle		
	Pearson's correlation	P-value	N
Clinical carrying angle	1		140
Age	0.970	<0.001**	140
BMI	-0.398	<0.001**	140
Average arm length	0.852	<0.001**	140
Average forearm length	0.884	<0.001**	140
Average inter-epicondylar distance	.852	<0.001**	140
Trans-trochanteric distance	0.845	<0.001**	140
Average Baumann’s angle	0.382	<0.001**	120

On multivariate linear regression, the CCA was found to be associated with age and inter-epicondylar distance (Table 5).

**Table 5:** Multivariable linear regression with the clinical carrying angle as the dependent variable.

Model	Unstandardized coefficients		Standardized coefficients	t	P-value
	B	Std. Error	Beta		
(Constant)	6.51	0.859		7.58	0
Age	0.445	0.03	1.047	15.069	0
BMI	-0.028	0.017	-0.04	-1.662	0.099
Average arm length (cm)	-0.01	0.034	-0.028	-0.293	0.77
Average forearm length (cm)	0.038	0.039	0.103	0.963	0.337
Inter-epicondylar distance (cm)	-0.278	0.074	-0.221	-3.762	0
Trans-trochanteric distance (cm)	0.01	0.013	0.037	0.784	0.434
Secondary sexual characteristics	0.033	0.165	0.006	0.202	0.84
Baumann’s angle	0.012	0.01	0.027	1.281	0.202

## Discussion

This study provides new information regarding the factors associated with CCA in the human elbow. It demonstrated a significant positive association between age and CCA and a significant negative association between CCA and inter-epicondylar distance. It is probably the first study to provide quantitative estimates of the association between age and CCA and that between inter-epicondylar distance and CCA. The un-standardized coefficient, B1, for age was 0.445, which means that for each one-year increase in age, there will be an increase in CCA by 0.445 degrees. The un-standardized coefficient, B5, for inter-epicondylar distance (cm) is equal to -0.278, which means that for each increase in inter-epicondylar distance (cm), there will be a decrease in the CCA by -0.2780 degrees. The present study is probably the first of its kind to use multivariable regression to describe the combined effect of multiple factors known to be correlated with carrying angle. Lastly, it corroborates the correlation between carrying angle and other morphometric factors. The present study did not find any statistically significant difference in the CCA and RCA in our study population, which is similar to the findings of a study done on Brazilian children up to the age of 16 years [22].

Several authors have investigated the effect of sex, age, and morphometric characteristics on the carrying angle. We did not find the CCA to be significantly higher in females than in males. However, several studies have reported the carrying angle to be higher in females compared to males [18, 19, 23]. It has been reported that a significantly higher carrying angle in females is seen at the age of nine years and continues till stabilization [22]. On the other hand, several researchers have not found any difference in the carrying angle irrespective of the sex and the age group [24, 25]. The greater carrying angle in females than males is considered a secondary sexual character [18]. The lack of significant difference in the carrying angle between males and females in the present study may be explained by the fact that in our study population, 113 (81.1%) were children below the age of 10 years, i.e., before the development of secondary sexual characteristics. The present study has reported a significant positive correlation between the CCA and increasing age. A positive association between age and carrying angles has been reported by other studies as well [5, 6, 22]. Balasubramanian P et al. [6] reported a strong correlation between the carrying angle and age up to 15 years, followed by a slight decrease. Terra BB et al. reported an increase in carrying angle up to the age of 16, after which it stabilized [22]. 

Several authors have investigated the relationship of morphometric factors (BMI, forearm length, arm length, inter-epicondylar distance, and trans-trochanteric diameter) on the carrying angle. Studies have reported a positive correlation between BMI and carrying angle [26,27]. In the present study, the CCA was found to be negatively correlated with BMI. However, the significance was lost on multivariable regression analysis. Similar to our findings, other researchers have also not found a correlation between BMI and carrying angle [28]. The difference may be explained based on the different ethnicities of the study population. There is contrasting evidence in the literature regarding the correlation of forearm length with carrying angle. A study that enrolled rural children from south India reported that carrying angle does not correlate with arm length or forearm length [6]. In contrast, a study conducted on MBBS students reported an inverse relationship between carrying angle and lengths of arm and forearm [13]. The present study reports a positive correlation between CCA and arm length and between carrying angle and forearm length. However, the significance was lost on multivariable regression.

An inverse relationship between inter-epicondylar distance and carrying angle has been reported in a study by Allouh MZ et al. that compared the difference in carrying angles of Jordanian and Malaysian children [15]. The inter-epicondylar distance was found to be negatively correlated with the CCA in our study. An important finding of our study is that the association was significant in multivariable regression. Ossification centers for the capitulum and medial epicondyle appear much earlier than the trochlea. As the capitulum ossifies, it grows medially, leaving limited space for the trochlear epiphysis [29]. As the trochlea ossifies, it grows distally to compensate for the limited space available, leading to the formation of the carrying angle [4]. The present study reports a significant positive correlation between trans-trochanteric diameter and the CCA. A significant positive correlation between the hip circumference and carrying angle has been reported by other studies as well [4,30]. However, the association was lost on multivariable regression.

Results of multivariable linear regression analysis in the present study show that age and inter-epicondylar distance are the only independent factors associated with CCA in the pediatric population.

Limitations of the study

We did not plan for a subgroup analysis based on the gender of the enrolled subjects, which is a limitation of our study. The factors associated with male carrying angles and female carrying angles may be different on account of hormonal factors. We recommend another study to identify factors independently associated with CCAs in males and females. 

The carrying angle in the dominant arm has been reported to be higher than in the non-dominant arm. Most of the children show clear-cut dominance by the age of three years. Since we did not want to exclude children below the age of three years, we did not investigate the effect of hand dominance on the carrying angle. We recommend that another study investigate the effect of hand dominance on carrying angle in the pediatric population. 

We did not investigate the role of ethnicity in carrying angle. The present study was done at a center that does not cater to subjects of different ethnicities. A future multi-centric study enrolling children of diverse ethnicities may help us resolve the effect of ethnicity on the carrying angle of children.

## Conclusions

This study demonstrated the role of age and inter-epicondylar distance as the only true factor associated with the CCA in children below 15 years. Although several morphometric factors like arm length, forearm length, Baumann's angle, and trans-trochanteric diameter correlate with carrying angle, we cannot consider them as true associations of CCA.

## References

[1] Snell RS (2004). Clinical Anatomy, 7th ed.

[2] Khare GN, Goel SC, Saraf SK, Singh G, Mohanty C (1999). New observations on carrying angle. Indian J Med Sci.

[3] Zampagni ML, Casino D, Martelli S, Visani A, Marcacci M (2008). A protocol for clinical evaluation of the carrying angle of the elbow by anatomic landmarks. J Shoulder Elbow Surg.

[4] Paraskevas G, Papadopoulos A, Papaziogas B, Spanidou S, Argiriadou H, Gigis J (2004). Study of the carrying angle of the human elbow joint in full extension: a morphometric analysis. Surg Radiol Anat.

[5] Golden DW, Jhee JT, Gilpin SP, Sawyer JR (2007). Elbow range of motion and clinical carrying angle in a healthy pediatric population. J Pediatr Orthop B.

[6] Balasubramanian P, Madhuri V, Muliyil J (2006). Carrying angle in children: a normative study. J Pediatr Orthop B.

[7] Cain EL Jr, Dugas JR, Wolf RS, Andrews JR (2003). Elbow injuries in throwing athletes: a current concepts review. Am J Sports Med.

[8] Hutchinson MR, Wynn S (2004). Biomechanics and development of the elbow in the young throwing athlete. Clin Sports Med.

[9] Van Roy P, Baeyens JP, Fauvart D, Lanssiers R, Clarijs JP (2005). Arthro-kinematics of the elbow: study of the carrying angle. Ergonomics.

[10] Robinson PM, Griffiths E, Watts AC (2017). Simple elbow dislocation. Shoulder Elbow.

[11] Chang CW, Wang YC, Chu CH (2008). Increased carrying angle is a risk factor for nontraumatic ulnar neuropathy at the elbow. Clin Orthop Relat Res.

[12] Sharma K, Mansur DI, Khanal K, Haque MK (2013). Variation of carrying angle with age, sex, height and special reference to side. Kathmandu Univ Med J (KUMJ).

[13] Kothapalli J, Murudkar PH, Seerla LD (2013). The carrying angle of elbow - A correlative and comparative study. Int J Cur Res Rev.

[14] Hassan D, Hossein S, Rahmani P, Hossein NS (2014). The study of predictor’s anthropometric parameters of upper limb with elbow carrying angle in athletes. Sports Med J.

[15] Allouh MZ, Abu Ghaida JH, Jarrar AA, Khasawneh RR, Mustafa AG, Bashaireh KM (2016). The carrying angle: racial differences and relevance to inter-epicondylar distance of the humerus. Folia Morphol (Warsz).

[16] Dai L (1999). Radiographic evaluation of Baumann angle in Chinese children and its clinical relevance. J Pediatr Orthop B.

[17] Mohammad S, Rymaszewski LA, Runciman J (1999). The Baumann angle in supracondylar fractures of the distal humerus in children. J Pediatr Orthop.

[18] Bari W, Alam M, Omar S (2017). Goniometry of elbow carrying angle: a comparative clinical study on sexual dimorphism in young males and females. Int J Res Med Sci.

[19] Keats TE, Teeslink R, Diamond AE, Williams JH (1966). Normal axial relationships of the major joints. Radiology.

[20] Morrey BF, An KN (2019). Functional Evaluation of the Elbow. Morrey's The Elbow and Its Disorders (Fourth Edition).

[21] Smajic N, Smajic J, Sadic S, Jasarevic M, Ahmetovic-Djug J, Hodzic R (2013). Correlation between Bauman's and carrying angle in children with supracondylar fracture of humerus. Med Arch.

[22] Terra BB, Mello Silva BC, Bella H, Carvalho FD, Dobashi ET, Pinto JA, Ishida A (2011). Evolution of the carrying angle of the elbow: a clinical and radiographic study. Acta Ortop Bras.

[23] Rai J, Parkash S, Singhal V (1980). Carrying angle in Indian girls and boys. Ind J Orthop.

[24] Purkait R, Chandra H (2004). An anthropometric investigation into the probable cause of formation of 'carrying angle': a sex indicator. JIAFM.

[25] Steel FL, Tomlinson JD (1958). The 'carrying angle' in man. J Anat.

[26] Anibor E, Ojigho E, Ogbatorho T (2016). Correlation between body mass index and carrying angle among adolescents in Abraka, Nigeria. Int J For Med Invest.

[27] Ansari MA, Bhagat PS, Thokal D, Ganvir SD (2021). Evaluation of the carrying angle of the elbow joint in adolescents and its correlation with BMI, gender and dominant side. Int J Sci Res.

[28] Golden DW, Wojcicki JM, Jhee JT, Gilpin SL, Sawyer JR, Heyman MB (2008). Body mass index and elbow range of motion in a healthy pediatric population: a possible mechanism of overweight in children. J Pediatr Gastroenterol Nutr.

[29] Fader LM, Laor T, Eismann EA, Cornwall R, Little KJ (2014). MR imaging of capitellar ossification: a study in children of different ages. Pediatr Radiol.

[30] Chinweife KC, Ejimofor OC, Ezejindu DN (2014). Correlation of carrying angle of the elbow in full extension and hip-circumference in adolescents of Nnewi people in Anambra state. Int J Sci Res Pub.

